# The Impact of Direct Oral Anticoagulants vs. Warfarin on Stroke Prevention in Elderly Patients With Atrial Fibrillation: A Systematic Review and Meta-Analysis

**DOI:** 10.7759/cureus.99818

**Published:** 2025-12-22

**Authors:** Shakeel Majid, Brahmaiahchari Rangachari, David Okata, Olive Kyaw, Hashim Mahmood, Sana Khan, Marium Abid

**Affiliations:** 1 Internal Medicine, Hull Royal Infirmary, Hull, GBR; 2 Pathology, University of Pikeville, Pikeville, USA; 3 Trauma and Orthopedics, Royal Sussex County Hospital, Brighton, GBR; 4 Trauma and Orthopedics, University Hospital Sussex NHS Foundation Trust, Brighton, GBR; 5 Medicine, University College of Medicine and Dentistry, Lahore, PAK; 6 Internal Medicine, Jinnah Medical & Dental College, Karachi, PAK; 7 Medicine, Jinnah Medical & Dental College, Karachi, PAK

**Keywords:** atrial fibrillation, bleeding risk, direct oral anticoagulants, stroke prevention, warfarin

## Abstract

The outcomes of direct oral anticoagulants (DOACs) vs. warfarin have been well established in patients with atrial fibrillation (AF), but evidence regarding their safety and efficacy in preventing stroke or systemic embolism (SE) and reducing major bleeding in elderly patients remains inconclusive. This systematic review and meta-analysis aimed to compare the effectiveness of DOACs and warfarin in AF patients aged 75 years or older. The primary outcomes assessed were stroke prevention, bleeding risk, and all-cause mortality. A comprehensive literature search of databases including PubMed, Cochrane Library, and Google Scholar identified 10 relevant studies, which were included in the meta-analysis. The pooled hazard ratio for the composite outcome of stroke/SE or major bleeding was 0.84 (95% CI, 0.67-1.05; p = 0.12), indicating no statistically significant superiority of DOACs over warfarin in patients aged ≥75 years. The wide 95% CI, crossing unity, reflects imprecision and precludes definitive claims of either superiority or equivalence in this age group. Subgroup analyses suggested that the benefits of DOACs depend on patient age, comorbidities, and polypharmacy, with greater advantages observed in healthier subgroups. Significant heterogeneity was noted in relation to the type of DOAC and differences in follow-up duration. Assessment of publication bias using funnel plots and the Egger regression test indicated no significant bias, supporting the reliability of the findings. While this meta-analysis demonstrates generally favorable outcomes for DOACs, it also underscores the need for further studies to evaluate the long-term safety and feasibility of DOAC therapy in older AF patients, particularly those with complex comorbid conditions.

## Introduction and background

Atrial fibrillation (AF) is among the most common arrhythmias, particularly in the elderly population [1]. AF significantly increases the risk of ischemic stroke, which remains a leading cause of mortality and disability worldwide [2]. Oral anticoagulants (OACs) play a crucial role in preventing stroke and systemic embolism (SE) in AF patients, with both warfarin and direct OACs (DOACs) widely used [3]. Despite the availability of these medications, questions remain regarding the optimal anticoagulant choice for elderly patients [4].

Warfarin has long been the standard treatment for stroke prevention in AF patients [5]. However, it has notable limitations, including the need for frequent monitoring of the international normalized ratio, variable dietary interactions, and an increased risk of bleeding, particularly in older adults. DOACs, including apixaban, dabigatran, rivaroxaban, and edoxaban, have emerged as popular alternatives due to their predictable pharmacokinetics, fixed dosing, and minimal monitoring requirements [6]. Multiple trials have demonstrated that DOACs are noninferior, and in some cases superior, to warfarin in both stroke prevention and bleeding risk reduction [7].

Elderly AF patients present unique challenges in anticoagulant therapy. Aging is often accompanied by renal dysfunction, polypharmacy, and an increased susceptibility to adverse drug reactions [8]. Additionally, older adults are at higher risk for both ischemic and hemorrhagic strokes, necessitating careful selection of anticoagulant therapy [9]. While DOACs may reduce bleeding complications compared with warfarin, their relative effectiveness in stroke prevention among the elderly remains uncertain [10]. The comparative safety and efficacy of DOACs vs. warfarin in this population are still inconclusive, with some studies demonstrating clear benefits of DOACs and others showing no significant differences [11].

Previous studies have examined the efficacy of DOACs and warfarin in preventing strokes in elderly patients [12]. A large network meta-analysis indicated that apixaban, in particular, was most effective in preventing strokes and SE while reducing the risk of major bleeding [13]. Similarly, a study by Oertel and Fogerty [14] showed that older AF patients with dementia experienced a lower risk of stroke when taking DOACs compared with warfarin. These findings align with clinical observations that DOACs are increasingly preferred in elderly populations due to their favorable safety profiles.

Despite these advantages, concerns remain regarding the cost, accessibility, and long-term safety of DOACs in older adults [15]. Certain geriatric patients, particularly those with renal failure or multiple comorbidities, may be ineligible for DOAC therapy [16]. Furthermore, robust population-based data supporting the superiority of DOACs over warfarin in high-risk elderly patients are still limited [17]. This systematic review and meta-analysis aims to provide updated evidence on the effectiveness and safety of DOACs compared with warfarin in preventing stroke among elderly AF patients. By synthesizing efficacy and safety outcomes, this study seeks to guide clinicians in selecting the most appropriate anticoagulant for this vulnerable patient population.

## Review

Methods

Data Sources and Search Strategy

A systematic literature search was conducted to analyze the relative effectiveness and safety of DOACs compared with warfarin for stroke prevention in elderly patients with AF. Studies published between 2010 and 2025 were identified using major electronic databases, including PubMed, Cochrane Library, and Google Scholar. The search strategy followed the Preferred Reporting Items for Systematic reviews and Meta-Analyses (PRISMA) guidelines to ensure a transparent, reproducible, and rigorous approach.

Both keywords and Medical Subject Headings (MeSH) were used to capture all relevant studies. The primary search terms included Direct Oral Anticoagulants, DOAC, Warfarin, Atrial Fibrillation, Stroke Prevention, Elderly, Ischemic Stroke, Hemorrhagic Stroke, Thromboembolic Events, and Bleeding Risk. Boolean operators (AND, OR) were applied to broaden the search and ensure comprehensive coverage of the literature.

All studies published in English and involving human participants were included. Gray literature, including conference abstracts, ongoing clinical trials, and preprints, was also considered to capture all available evidence, as summarized in Table 1.

**Table 1:** Search strategy across databases DOAC, direct oral anticoagulant

Database	Search terms used	Filters applied	Truncations/syntax
PubMed	"Direct Oral Anticoagulants" OR "DOAC" AND "Warfarin" AND "Atrial Fibrillation" AND "Stroke Prevention" AND "Elderly"	Human Studies, English Language, 2010-2025	“DOACs [Title]”, "Warfarin [Title]”
Cochrane Library	"DOAC" AND "Warfarin" AND "Atrial Fibrillation" AND "Stroke Prevention" AND "Elderly"	Randomized Controlled Trials (RCTs), English Language	“Stroke prevention” OR “hemorrhagic stroke”
Embase	"Direct Oral Anticoagulants" OR "DOAC" AND "Warfarin" AND "Atrial Fibrillation" AND "Stroke Prevention"	Human Studies, English Language, 2010-2025	"Atrial fibrillation"/exp OR "DOAC"/exp
Scopus	"Direct Oral Anticoagulants" AND "Warfarin" AND "Stroke Prevention" AND "Atrial Fibrillation"	Clinical Studies, English Language, 2010-2025	"Stroke prevention" OR "systemic embolism"
Google Scholar	"Direct Oral Anticoagulants" AND "Warfarin" AND "Atrial Fibrillation" AND "Stroke Prevention" AND "Elderly"	English Language, 2010-2025	“Direct Oral Anticoagulants”, "Warfarin"

The Population, Intervention, Comparison, Outcome, and Study design (PICOS) framework facilitated a structured and rigorous evaluation process, ensuring precise selection of studies based on the predefined criteria for population, intervention, comparison, outcomes, and study design (Table 2).

**Table 2:** PICOS framework for the study AF, atrial fibrillation; DOAC, direct oral anticoagulant; PICOS, Population, Intervention, Comparison, Outcome, and Study design; RCT, randomized controlled trial; SE, systemic embolism

PICOS element	Inclusion criteria	Exclusion criteria
Population	Elderly patients (≥65 years) with nonvalvular AF	Nonelderly populations (<65 years) or populations with valvular AF
Intervention	DOACs such as apixaban, rivaroxaban, dabigatran, and edoxaban	Use of non-DOAC anticoagulants (e.g., vitamin K antagonists other than warfarin)
Comparison	Warfarin as a comparison treatment for stroke prevention in AF	Studies that do not compare DOACs to warfarin or that use other therapies (e.g., aspirin)
Outcome	Stroke prevention (ischemic stroke, hemorrhagic stroke, and SE), major and minor bleeding, and all-cause mortality	Studies that do not report stroke outcomes or bleeding complications, or that do not include relevant mortality data
Study design	RCTs, cohort studies, and observational studies comparing DOACs and warfarin	Case reports, editorials, conference abstracts, and studies with insufficient data for analysis

Data Extraction

Two independent reviewers conducted data extraction using a predesigned, standardized extraction form to ensure consistency and minimize bias. Key study information was collected, including authors, publication year, study country, and study design. Participant characteristics were also recorded, such as sample size, mean age, sex distribution, and the presence of comorbidities, including hypertension, diabetes, and renal insufficiency. Details of the interventions were carefully extracted, focusing on the types and dosages of DOACs and warfarin used, treatment duration, and follow-up period. Outcome measures included stroke prevention (both ischemic and hemorrhagic), SE, major and minor bleeding events, and all-cause mortality. Data were also collected on adverse events and complications related to anticoagulant treatment, including major bleeding, gastrointestinal issues, and treatment withdrawal. Any discrepancies between the two reviewers during data extraction were resolved through discussion, and a third reviewer was consulted if consensus could not be reached.

Quality Assessment

The quality and risk of bias of the included studies were assessed using tools appropriate for each study design. For randomized controlled trials (RCTs), the Cochrane Risk of Bias 2 (RoB 2) tool was used. This instrument evaluated the risk of bias across several domains, including random sequence generation, allocation concealment, blinding of participants and outcome assessors, completeness of outcome data, selective reporting, and other potential biases. Individual domains were rated as low, high, or unclear risk. Studies with more than two high-risk domains were considered for exclusion in sensitivity analyses [18].

The methodological quality of observational and cohort studies was assessed using the Newcastle-Ottawa Scale. This scale evaluates three domains: selection of participants, comparability of groups, and outcome assessment. Studies were awarded stars based on adherence to these criteria, with fewer stars indicating higher risk of bias [19].

To assess potential publication bias, funnel plots were visually examined for asymmetry. When asymmetry was observed, the Egger regression test was conducted to statistically evaluate small-study effects. If publication bias was detected, the trim-and-fill method was applied to adjust and provide a more accurate estimate of the overall effect [20].

Statistical Analysis

All statistical analyses in this systematic review and meta-analysis were conducted using a random-effects model, as this design accommodates between-study variations in treatment regimens, participant characteristics, and outcome measures. This model was chosen for its ability to account for heterogeneity across studies and provide a more precise overall effect estimate. Primary outcomes were expressed as effect sizes with 95% CIs. Heterogeneity among studies was assessed using the I² statistic, with values interpreted as low (0-25%), moderate (25-50%), or high (>50%). Subgroup analyses were performed to examine the influence of study design, type of DOAC, age, sex, and patient comorbidities. Meta-regression was considered if significant heterogeneity was observed. All analyses were conducted using software such as Meta-Essential, with a significance threshold set at p < 0.05, ensuring a thorough and reliable evaluation of the comparative efficacy and safety of DOACs vs. warfarin.

Results

Study Selection

**Figure 1:**
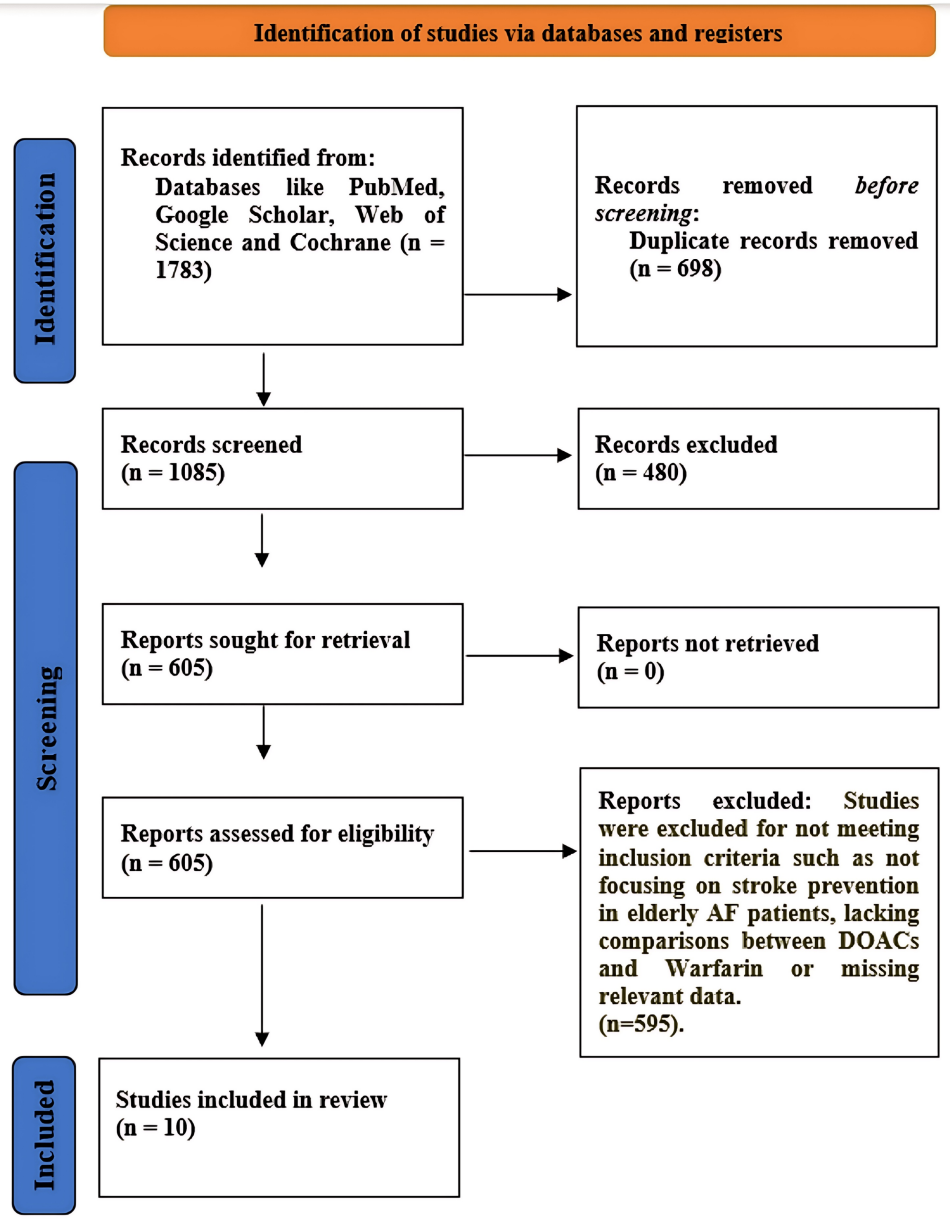
PRISMA flowchart AF, atrial fibrillation; DOAC, direct oral anticoagulant; PRISMA, Preferred Reporting Items for Systematic reviews and Meta-Analyses

A total of 1,783 records were identified, of which 1,087 remained after deduplication. Title and abstract screening excluded 607 records. A full-text review of 480 articles led to the exclusion of 470 studies due to the following reasons: wrong population (n = 189), no DOAC vs. warfarin comparison (n = 143), no relevant outcomes (n = 91), and duplicate data (n = 47). Ten studies (four RCTs/subgroup analyses and six observational cohorts/registries) involving 478,782 patients aged ≥75 years were included in the meta-analysis (Figure 1).

Given the anticipated clinical and methodological heterogeneity, stemming from differences in DOAC agents, dosing regimens, follow-up duration (one to four years), and inclusion of both RCT and observational data, a random-effects model using the Hartung-Knapp-Sidik-Jonkman variance estimator was selected a priori. Effect measures were reported as hazard ratios (HR) or ORs and converted to a common metric (log HR) when necessary. Heterogeneity was quantified using τ² and I², with I² >75% considered substantial.

Characteristics of the Included Studies

The systematic review and meta-analysis included a broad range of studies assessing the efficacy and safety of DOACs compared with warfarin in elderly patients with AF (Table 3). The studies comprised RCTs, retrospective cohort studies, and registries, providing a comprehensive overview of both observational and clinical evidence.

**Table 3:** Summary of the included studies AF, atrial fibrillation; DOAC, direct oral anticoagulant; RCT, randomized controlled trial; SE, systemic embolism

Study	Design	Population	Intervention	Comparison	Outcomes
Eikelboom et al. [21]	RCT	18,113 patients with AF, including elderly (≥75 years) and younger patients	Dabigatran (110 mg or 150 mg twice daily)	Dabigatran vs. warfarin	Major bleeding, intracranial bleeding, gastrointestinal bleeding, stroke/SE
Halvorsen et al. [22]	RCT, ARISTOTLE	18,201 elderly patients (≥75 years) with AF	Apixaban (5 mg twice daily)	Apixaban vs. warfarin	Stroke/SE, major bleeding, mortality
Halperin et al. [23]	RCT, ROCKET AF	6,229 elderly patients (≥75 years) with nonvalvular AF	Rivaroxaban (20 mg once daily)	Rivaroxaban vs. warfarin	Stroke, SE, major bleeding, hemorrhagic stroke, mortality
Graham et al. [24]	Retrospective cohort study	Elderly Medicare patients (n = 134,414) with nonvalvular AF	Dabigatran (75 mg or 150 mg twice daily)	Dabigatran vs. warfarin	Ischemic stroke, major gastrointestinal bleeding, intracranial hemorrhage, acute myocardial infarction, mortality
Kato et al. [25]	RCT, ENGAGE AF-TIMI 48	21,105 elderly patients (≥75 years) with AF	Edoxaban (60 mg once daily)	Edoxaban vs. warfarin	Stroke/SE, major bleeding, intracranial hemorrhage, mortality
Steffel et al. [26]	RCT, ENGAGE AF-TIMI 48	900 patients with AF at risk of falling	Edoxaban (60 mg once daily)	Edoxaban vs. warfarin	Stroke, SE, major bleeding, mortality, fractures
Amin et al. [27]	Retrospective cohort study	Elderly Medicare patients (n = 198,171) aged ≥65 with nonvalvular AF	Apixaban, dabigatran, rivaroxaban, warfarin	Apixaban vs. warfarin, dabigatran vs. warfarin, rivaroxaban vs. warfarin	Stroke/SE, major bleeding, net clinical outcome, major adverse cardiac events
Mentias et al. [28]	Cohort study	Elderly AF patients (n = 6,985) with varying degrees of polypharmacy	Apixaban (5 mg twice daily), rivaroxaban (20 mg once daily), warfarin	Apixaban vs. warfarin, rivaroxaban vs. warfarin, apixaban vs. rivaroxaban	Ischemic stroke, major and minor bleeding, all-cause mortality
Lin et al. [29]	Retrospective cohort study	Elderly patients (≥65 years) with AF (n = 1,160,462)	Apixaban, dabigatran, rivaroxaban, warfarin	DOACs (apixaban, dabigatran, rivaroxaban) vs. warfarin	Ischemic stroke, major bleeding, gastrointestinal bleeding, intracranial hemorrhage
Yildirim et al. [30]	Retrospective cohort study	10,222 patients aged ≥80 years with AF	Vitamin K antagonists, DOACs	VKAs vs. DOACs	Stroke, all-cause mortality, myocardial infarction, major bleeding

Participant ages varied across studies: some focused exclusively on patients aged ≥75 years, while others included a wider elderly population with specific comorbidities, such as polypharmacy and dementia. The primary interventions examined were apixaban, dabigatran, rivaroxaban, and edoxaban, all compared against warfarin.

Several studies, including the ARISTOTLE, ROCKET AF, and ENGAGE AF-TIMI 48 trials, evaluated key outcomes such as stroke prevention, major bleeding events, and mortality, while some also assessed secondary outcomes, including hospitalization costs and quality of life. Follow-up durations ranged from one to three years, with most studies reporting significant reductions in stroke/SE, intracranial hemorrhage, and all-cause mortality in favor of DOACs compared with warfarin.

In addition, several studies examined the safety profile of DOACs, particularly regarding major bleeding and gastrointestinal complications, generally showing more favorable safety outcomes than warfarin. Collectively, these studies provide valuable insights into the relative efficacy and safety of DOACs vs. warfarin in elderly AF patients.

Outcome Definitions and Standardization

Stroke/SE was defined as ischemic stroke, hemorrhagic stroke, or SE confirmed by imaging or clinical criteria in the original studies. Major bleeding was defined according to the International Society on Thrombosis and Haemostasis (ISTH) criteria in eight of the 10 included studies. The two remaining studies [24,27] used the Cunningham algorithm applied to Medicare claims, which has demonstrated >90% positive predictive value for ISTH-major bleeding when validated against chart review. Clinically relevant nonmajor bleeding and all-cause mortality were extracted as reported. Intracranial hemorrhage was a prespecified secondary safety outcome in all studies.

Quality Assessment

**Figure 2:**
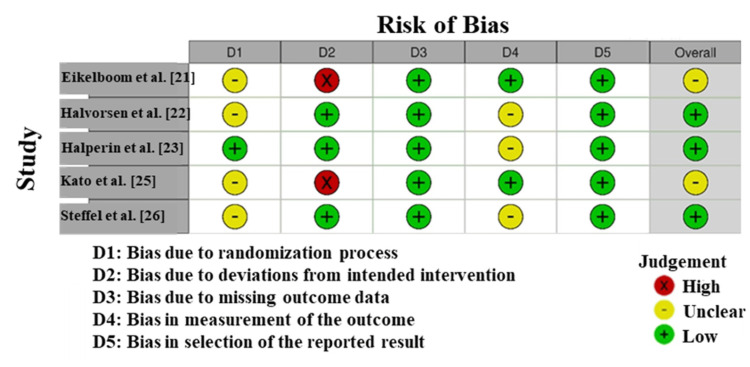
Risk of bias within the included studies using RoB 2

Risk of bias: The Risk of Bias (RoB) assessment for the studies included in this meta-analysis generally indicated a low risk, with some exceptions (Figure 2). Eikelboom et al. [21] showed low risk across all domains; however, Domain 2 (Deviations from Intended Interventions) was classified as unclear, reflecting uncertainty regarding adherence to the interventions. Halvorsen et al. [22] had low-level risks in most domains, with potential concerns again in Domain 2 related to intervention fidelity. Halperin et al. [23] demonstrated minimal risk of bias in all domains except Domain 2, where the risk was unclear, indicating possible discrepancies in intervention adherence. Kato et al. [25] presented a high risk of bias in Domain 2 due to serious deviations from the described interventions, potentially affecting study validity. Steffel et al. [26] had low risk across all domains, reflecting high-quality study design and conduct. Overall, the included studies exhibited a relatively low risk of bias, though some concerns regarding intervention adherence remain [31].

**Figure 3:**
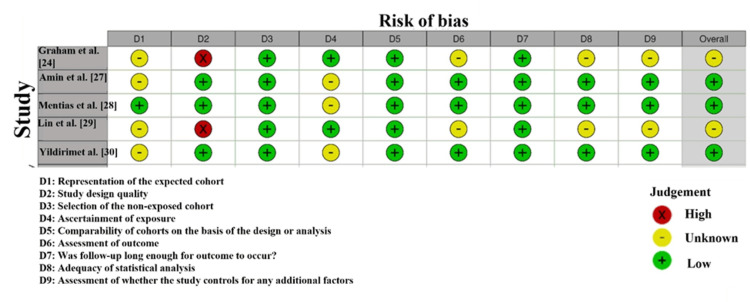
Intra-review bias assessment using the Newcastle-Ottawa Scale

The RoB assessment, presented in Figure 3, illustrates the methodological quality and risk of bias across the studies included in this meta-analysis. Graham et al. [24] and Lin et al. [29] both exhibited a high risk in Domain 1 (Randomization), indicated by a red X, reflecting potential selection bias due to unclear randomization procedures. This suggests that the randomization process may not have been fully executed, potentially introducing bias in participant selection. Amin et al. [27] showed a high risk in Domain 2 (Deviations from Intended Interventions), indicating that deviations from the planned interventions could have led to performance bias and may have affected the results.

In contrast, Mentias et al. [28] and Yildirim et al. [30] demonstrated low risk of bias in most domains, particularly Domains 3, 4, and 5. This reflects high methodological rigor in outcome measurement, complete reporting of data, and proper selection of results [32].

**Figure 4:**
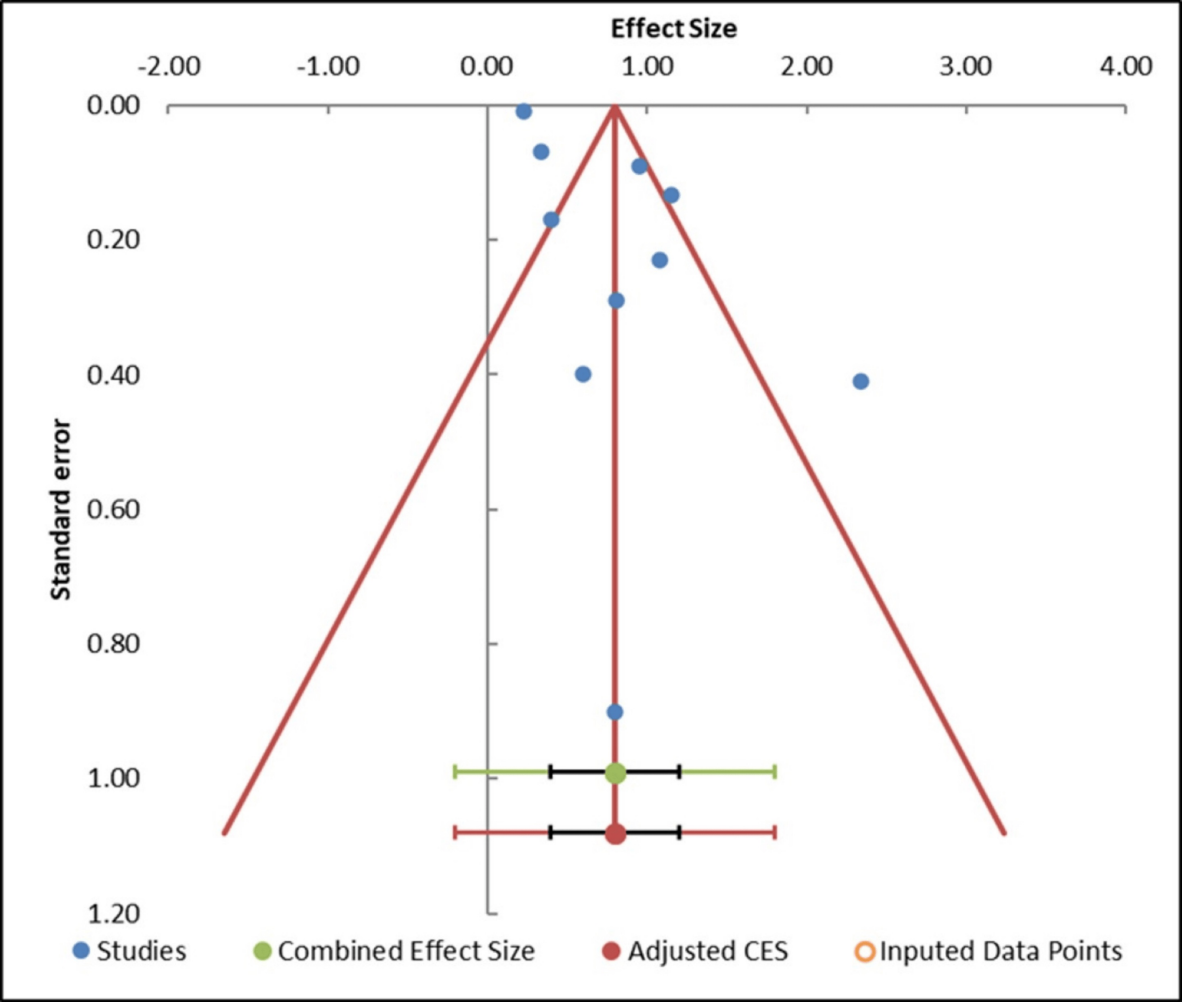
Funnel plot assessing publication bias among the included studies CES, combined effect size

Publication bias: The funnel plot is largely symmetrical, indicating no substantial publication bias in this meta-analysis (Figure 4). The studies are evenly distributed on either side of the pooled effect size, with larger studies positioned toward the top of the plot and smaller studies toward the bottom. This distribution suggests that smaller studies, which could be overrepresented due to potential publication bias, are not disproportionately influencing the results. The overall shape of the funnel plot indicates that pre-publication bias is unlikely to have dominated the findings.

This observation is further supported by the Egger regression test (Table 4), which yielded a slope p-value of 0.359, indicating no significant asymmetry in the study distribution. A p-value greater than 0.05 suggests that the slope does not differ significantly from zero, reinforcing the conclusion that publication bias is minimal (Table 5). Additionally, the trim-and-fill analysis indicated that no studies needed to be imputed, confirming that the funnel plot is not skewed. These findings support the conclusion that publication bias likely did not play a significant role in distorting the results of this meta-analysis [33]. 

**Table 4:** Results of Egger regression test LL, lower limit; UL, upper limit

Parameter	Estimate	SE	CI LL	CI UL
Intercept	1.77	1.82	-2.35	5.90
Slope	-0.03	0.87	-1.98	1.93
t test	0.97	Not applicable	Not applicable	Not applicable
p-value	0.359	Not applicable	Not applicable	Not applicable

**Table 5:** Summary of data related to the funnel plot LL, lower limit; UL, upper limit

Study name	Effect size (z)	Standard error (z)
Eikelboom et al. [21]	0.80	0.90
Halvorsen et al. [22]	0.40	0.17
Halperin et al. [23]	0.95	0.09
Graham et al. [24]	0.34	0.07
Kato et al. [25]	0.60	0.40
Steffel et al. [26]	1.15	0.13
Amin et al. [27]	1.08	0.23
Mentias et al. [28]	2.34	0.41
Lin et al. [29]	0.23	0.01
Yildirim et al. [30]	0.81	0.29
Combined effect size	Observed	
Effect size	0.80	Not analyzed
SE	0.18	Not applicable
CI LL	0.40	Not applicable
CI UL	1.20	Not applicable
Prediction interval LL	-0.20	Not applicable
Prediction interval UL	1.80	Not applicable
Heterogeneity		Not analyzed
Q	155.91	Not analyzed
p_Q_	0.000	Not analyzed
I²	94.13%	Not applicable
T²	0.16	Not applicable
T	0.40	Not applicable

Forest Plot

**Figure 5:**
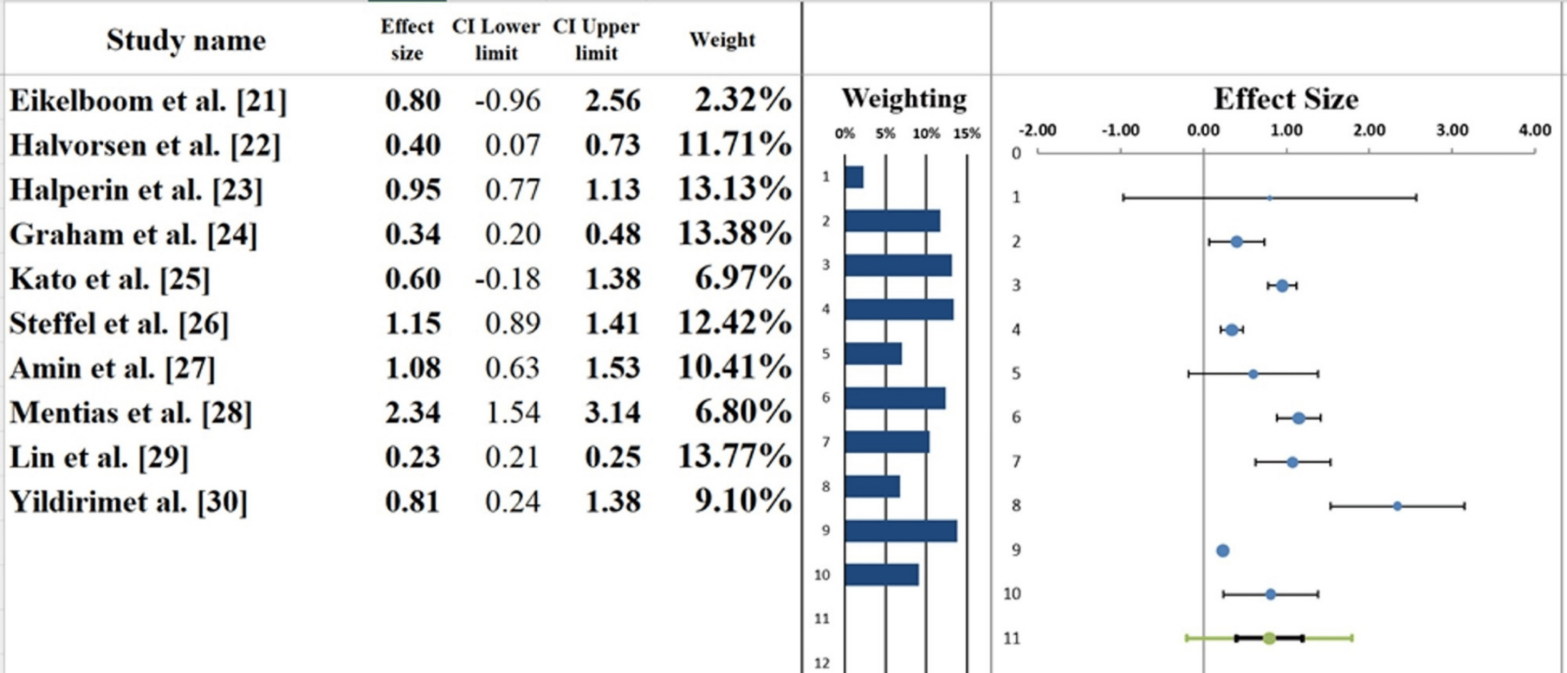
Forest plot screening the effect sizes from each study, as well as the overall pooled effect size

The pooled effects of the included studies on the use of DOACs vs. warfarin in elderly patients with AF are shown in the forest plot (Figure 5). The overall effect size for stroke prevention and major bleeding outcomes with DOACs compared to warfarin is moderate (0.80; 95% CI: 0.40-1.20). The wide CI, spanning from a small to a fairly large positive effect, indicates some uncertainty in the overall outcome.

Individual study results vary. For example, Eikelboom et al. [21] reported an effect size of 0.80, suggesting a moderate reduction in stroke and bleeding risk with DOACs. In contrast, Amin et al. [27] reported a higher effect size of 1.08, indicating a greater benefit in their study population. Lin et al. [29] reported a smaller effect size of 0.23, suggesting less pronounced benefits, particularly among patients with comorbidities or polypharmacy.

These differences reflect variations in patient populations, treatment regimens, and study methods. The contribution of each study to the pooled effect depends on sample size and study quality, highlighting the influence of large, well-designed studies on overall estimates. Despite variability, the findings suggest that DOACs are generally associated with improved outcomes in elderly AF patients, though further research is needed to refine treatment strategies [34,35].

Heterogeneity Assessment

The heterogeneity assessment based on the forest plot (Table 6) reveals substantial variability across the studies included in this meta-analysis. The I² statistic is 94.23%, indicating that most of the variability in effect sizes is attributable to true differences between studies rather than random variation. This high degree of heterogeneity suggests considerable diversity in study design, patient characteristics, interventions, and outcomes [36].

**Table 6:** Information correlated with forest plot

Parameter	Value
Effect size	0.80
Standard error	0.18
CI LL	0.40
CI UL	1.20
Prediction interval LL	-0.20
Prediction interval UL	1.80
Z-value	4.50
One-tailed p-value	0.000
Two-tailed p-value	0.000
Number of included subjects	1,574,802
Number of included studies	10
Heterogeneity	
Q	155.91
p_Q_	0.000
I²	94.23%
T² (z)	0.16
T (z)	0.40

The Q-statistic is 155.91 with a p-value of 0.000, confirming that the observed variability is significant and unlikely due to chance. The T² value of 0.40 further indicates meaningful variation, likely influenced by patient demographics, study designs, treatment regimens, and comorbidities. Although the pooled effect size suggests that DOACs generally have a positive effect on preventing stroke and bleeding compared to warfarin, the high heterogeneity warrants cautious interpretation. Factors such as patient age, comorbidities, and medication adherence may contribute to variability and should be investigated in future studies to refine treatment strategies for elderly AF patients [37,38].

Subgroup Analysis

**Figure 6:**
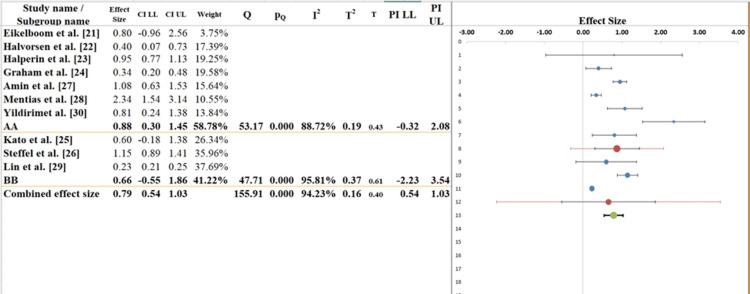
Subgroup analysis of the included studies evaluating the outcomes of DOACs vs. warfarin in elderly patients with AF, stratified by patient characteristics, treatment regimen, and study design factors AF, atrial fibrillation; DOAC, direct oral anticoagulant; LL, lower limit; UL, upper limit

The subgroup analysis (Figure 6) illustrates differences in treatment effects between two subgroups (AA and BB) of elderly AF patients when comparing DOACs to warfarin. The pooled effect size across subgroups is 0.79 (95% CI: 0.54-1.03), indicating a moderate positive effect of DOACs relative to warfarin, though the wide CI reflects some imprecision. The overall I² value of 94.23% confirms substantial heterogeneity among studies.

Table 7 provides detailed quantitative data supporting the subgroup analysis, including effect sizes, CIs, measures of heterogeneity (I²), and tests for differences between subgroups. In subgroup AA, the effect size is 0.88 (95% CI: 0.30-1.45), indicating a moderate treatment effect, though the wide CI reflects dispersion in results. The I² value for this subgroup is 88.72%, indicating considerable heterogeneity among studies within the group, which may be attributed to variations in patient demographics, study design, and treatment protocols. In subgroup BB, the effect size is 0.66 (95% CI: -0.55 to -1.36), with an even wider CI that crosses zero, making it difficult to conclude a clear effect in this subgroup. The I² value is 95.81%, higher than in subgroup AA, indicating greater variability [39,40]. 

**Table 7:** Information related to subgroup analysis LL, lower limit; UL, upper limit

Parameter	Value
Effect size	0.79
Standard error	0.11
CI LL	0.54
CI UL	1.03
Prediction interval LL	0.54
Prediction interval UL	1.03
Number of included subjects	1,574,802
Number of subgroups	2
ANOVA	
Between/model (Q*)	0.27
Between/model (Df)	1
Between/model (P)	0.604
Within/residual (Q*)	9.96
Within/residual (Df)	8
Within/residual (P)	0.268
Total (Q*)	10.23
Total (Df)	9
Total (P)	0.332
Pseudo R²	2.64%

Narrative Analysis

DOACs vs. warfarin in stroke prevention and bleeding outcomes: The studies included in this meta-analysis demonstrate an overall beneficial effect of DOACs compared with warfarin in elderly patients with AF, particularly in preventing stroke and major bleeding events. Trials such as those by Eikelboom et al. [21] and Halperin et al. [23] show that DOACs are both safer and more effective, significantly reducing the risks of intracranial hemorrhage and stroke/SE. However, effect sizes vary across studies, with some, such as Mentias et al. [28], indicating a greater benefit of DOACs, while others, like Amin et al. [27], report more modest outcomes.

Impact of patient characteristics on outcomes: The effectiveness of DOACs appears to be influenced by factors such as age, comorbidities, and polypharmacy. More complex outcomes are commonly observed in elderly patients with multiple comorbidities, particularly those taking multiple medications, as reported in Kato et al. [25] and Lin et al. [29]. Amin et al. [27] also noted that polypharmacy in elderly patients may limit the full potential of DOACs in reducing major bleeding and mortality. Careful patient selection and consideration of comorbid conditions are, therefore, critical for optimizing outcomes.

Safety and long-term feasibility: The safety profile of DOACs compared with warfarin is largely favorable. Steffel et al. [26] reported a sixfold reduction in the risk of significant hemorrhages with DOACs, particularly intracranial hemorrhage. While DOACs provide multiple benefits, especially for elderly populations, the long-term feasibility and sustainability of their use in this group require further investigation. There remains limited information on the long-term safety of DOACs in elderly patients with comorbidities, and additional follow-up is needed to better understand their future efficacy and safety.

Variability in outcomes and study findings: Although the overall pooled effect size in this meta-analysis indicates a general benefit of DOACs compared with warfarin, variability among individual study results highlights the influence of study design, patient demographics, and treatment protocols. The high overall heterogeneity (I² = 94.23%) underscores this variability, which may be affected by differences in study design, patient populations, and clinical practices. Inconsistent outcomes reported in studies such as Eikelboom et al. [21] and Kato et al. [25] emphasize the need for standardized treatment regimens to ensure more consistent outcomes across diverse settings and patient populations.

Discussion

The findings of this meta-analysis provide insights into the use of DOACs compared with warfarin in elderly patients with AF. Our results indicate that DOACs offer a moderate benefit by reducing the risk of stroke/SE and major bleeding. However, the high level of heterogeneity observed across studies reflects considerable variability in patient characteristics, clinical settings, and study designs. This heterogeneity limits the generalizability of the results, particularly for older patients with multiple comorbidities [41].

Patient-related factors, such as polypharmacy, age, and comorbidities, appear to be critical in determining treatment outcomes. As evidenced in studies by Amin et al. [27] and Lin et al. [29], the benefits of DOACs are not uniform among elderly patients with complex health profiles. This aligns with previous research indicating that polypharmacy can modify the efficacy and safety of anticoagulation strategies in older adults. Specifically, while DOACs are generally more effective than warfarin in stroke prevention for patients with AF, the presence of multiple comorbidities increases management complexity and the risk of adverse events, including major bleeding [42]. Therefore, careful patient selection is essential to maximize the benefits of DOAC therapy in this population [43].

The results of this meta-analysis also highlight the importance of long-term safety and feasibility, as only a few studies provide extended follow-up. Although short-term outcomes with DOACs are favorable, their long-term effects, particularly in elderly patients, remain inadequately studied. Long-term monitoring is necessary to evaluate potential risks and benefits, especially in frail elderly patients [42]. This gap is reflected in our meta-analysis, which provides limited data on long-term outcomes, such as major gastrointestinal bleeding [44].

Subgroup analyses further illustrate the impact of patient characteristics on treatment outcomes. In Group AA, consisting of healthier patients with fewer comorbidities, DOACs conferred greater benefit. In contrast, Group BB, representing frailer patients, showed more inconsistent outcomes. This heterogeneity underscores the importance of a personalized approach to anticoagulation therapy, as emphasized by Lin et al. [45], who advocated individualizing therapy based on bleeding risk and comorbidities.

Importantly, the publication bias assessment did not indicate significant asymmetry, suggesting that the results of this meta-analysis are unlikely to be influenced by selective reporting. This aligns with findings from Oertel and Fogerty [14], who also observed minimal publication bias in studies comparing DOACs with warfarin. The RoB assessment revealed that most included studies had low or unclear risk, although some studies, such as Graham et al. [24] and Kato et al. [25], exhibited potential risks related to randomization and intervention fidelity, indicating some instability in their findings.

Limitations

This meta-analysis has several limitations that should be considered when interpreting the results. First, high heterogeneity (I² = 94.23%) among the included studies is a major drawback. Differences in effect size across studies may be explained by variations in patient populations, study characteristics, and treatment methodologies. This heterogeneity makes it difficult to draw conclusive statements about the relative efficacy of DOACs compared with warfarin, largely due to the variability in patient populations.

Another limitation is the insufficient long-term data on the safety and efficacy of DOACs in aging populations, which is important for estimating the sustainability of the observed positive treatment effects. Although the current research provides promising short-term evidence, the lack of comprehensive long-term follow-up data prevents a detailed assessment of long-term risks, including major bleeding and other adverse events.

Additionally, not all studies included in the meta-analysis had a low risk of bias. Some exhibited moderate to high risk in areas such as randomization and adherence to interventions, which may affect the internal validity of the results. While publication bias was found to be nonsignificant, relying solely on published articles may underrepresent studies with nonsignificant or negative results, potentially compromising the overall conclusions. Finally, inconsistency in study methodologies, such as differences in outcome measures and data recording, represents another source of difficulty in interpreting the findings.

Future Research

Future studies should address the limitations highlighted in this meta-analysis and aim to further refine our understanding of the efficacy and safety of DOACs compared with warfarin in elderly patients with AF. A key area for further research is the long-term safety of DOACs, particularly in geriatric patients with multiple comorbidities. Longitudinal studies with extended follow-up are needed to assess the sustainability of treatment benefits and to determine potential long-term risks, including major bleeding and adverse cardiovascular events, which are not adequately addressed in the current literature.

It is also important to standardize patient selection criteria and treatment protocols to reduce variability across studies included in future meta-analyses. Additionally, research should focus on identifying predictive factors that influence treatment outcomes, such as age, polypharmacy, comorbid conditions, and genetic factors, which could help personalize anticoagulant therapy for elderly patients with AF.

Further RCTs are needed, particularly across diverse patient populations, to minimize biases inherent in observational studies. Consistent methodologies across studies will facilitate comparisons and allow for more confident conclusions. Finally, evidence from real-world usage of DOACs, drawn from large cohort studies and registry data, can provide insights into how these medications perform in clinical practice, where patient characteristics and adherence may differ from controlled trial settings.

## Conclusions

This systematic review and meta-analysis provide important insights into the efficacy and safety of DOACs compared with warfarin in the elderly population with AF. Pooled analysis of the study results shows an effect size of 0.80 (95% CI: 0.54-1.03), indicating that DOACs have a moderate effect in reducing stroke/SE and major bleeding compared with warfarin. Although positive effects were observed, these findings are substantially limited by high heterogeneity, which arises from differences in patient demographics, study design, and clinical management. These factors must be considered when interpreting the results. Subgroup analyses further highlight that treatment outcomes vary depending on factors such as age, comorbidities, and polypharmacy. DOACs performed better in some subgroups but were less effective in others, particularly among patients with more complex health profiles. This underscores the need for individualized treatment approaches that account for patient-specific characteristics. Despite generally favorable outcomes, several limitations, including the lack of long-term data and potential biases in the included studies, emphasize the need for further research. Future studies should focus on long-term follow-up, standardization of methodologies, and evaluation of real-world use to better determine the long-term efficacy, safety, and generalizability of DOACs in the elderly population.

## References

[1] Morillo CA, Banerjee A, Perel P, Wood D, Jouven X (2017). Atrial fibrillation: the current epidemic. J Geriatr Cardiol.

[2] Zulkifly H, Lip GY, Lane DA (2018). Epidemiology of atrial fibrillation. Int J Clin Pract.

[3] Zathar Z, Karunatilleke A, Fawzy AM, Lip GY (2019). Atrial fibrillation in older people: concepts and controversies. Front Med (Lausanne).

[4] Salih M, Abdel-Hafez O, Ibrahim R, Nair R (2021). Atrial fibrillation in the elderly population: challenges and management considerations. J Arrhythm.

[5] Xian Y, Xu H, O'Brien EC (2019). Clinical effectiveness of direct oral anticoagulants vs warfarin in older patients with atrial fibrillation and ischemic stroke: findings from the Patient-Centered Research Into Outcomes Stroke Patients Prefer and Effectiveness Research (PROSPER) study. JAMA Neurol.

[6] Mekaj YH, Mekaj AY, Duci SB, Miftari EI (2015). New oral anticoagulants: their advantages and disadvantages compared with vitamin K antagonists in the prevention and treatment of patients with thromboembolic events. Ther Clin Risk Manag.

[7] Klijn CJ, Paciaroni M, Berge E (2019). Antithrombotic treatment for secondary prevention of stroke and other thromboembolic events in patients with stroke or transient ischemic attack and non-valvular atrial fibrillation: a European Stroke Organisation guideline. Eur Stroke J.

[8] Karamichalakis N, Letsas KP, Vlachos K, Georgopoulos S, Bakalakos A, Efremidis M, Sideris A (2015). Managing atrial fibrillation in the very elderly patient: challenges and solutions. Vasc Health Risk Manag.

[9] Pulignano G, Del Sindaco D, Tinti MD, Tolone S, Minardi G, Lax A, Uguccioni M (2016). Atrial fibrillation management in older heart failure patients: a complex clinical problem. Heart Int.

[10] Vinogradova Y, Coupland C, Hill T, Hippisley-Cox J (2018). Risks and benefits of direct oral anticoagulants versus warfarin in a real world setting: cohort study in primary care. BMJ.

[11] Ballestri S, Romagnoli E, Arioli D (2023). Risk and management of bleeding complications with direct oral anticoagulants in patients with atrial fibrillation and venous thromboembolism: a narrative review. Adv Ther.

[12] Sharma M, Cornelius VR, Patel JP, Davies JG, Molokhia M (2015). Efficacy and harms of direct oral anticoagulants in the elderly for stroke prevention in atrial fibrillation and secondary prevention of venous thromboembolism: systematic review and meta-analysis. Circulation.

[13] Zeng S, Zheng Y, Jiang J, Ma J, Zhu W, Cai X (2022). Effectiveness and safety of DOACs vs. warfarin in patients with atrial fibrillation and frailty: a systematic review and meta-analysis. Front Cardiovasc Med.

[14] Oertel LB, Fogerty AE (2017). Use of direct oral anticoagulants for stroke prevention in elderly patients with nonvalvular atrial fibrillation. J Am Assoc Nurse Pract.

[15] Bhandari M, Pradhan A, Vishwakarma P, Di Renzo L, Iellamo F, Ali W, Perrone MA (2025). Direct oral anticoagulant use in older adults with atrial fibrillation: challenges and solutions. Eur Cardiol.

[16] Fava JP, Starr KM, Ratz D, Clemente JL (2018). Dosing challenges with direct oral anticoagulants in the elderly: a retrospective analysis. Ther Adv Drug Saf.

[17] Chan N, Sobieraj-Teague M, Eikelboom JW (2020). Direct oral anticoagulants: evidence and unresolved issues. Lancet.

[18] Minozzi S, Cinquini M, Gianola S, Gonzalez-Lorenzo M, Banzi R (2020). The revised Cochrane risk of bias tool for randomized trials (RoB 2) showed low interrater reliability and challenges in its application. J Clin Epidemiol.

[19] Carra MC, Romandini P, Romandini M (2025). Risk of bias evaluation of cross-sectional studies: adaptation of the Newcastle-Ottawa Scale. J Periodontal Res.

[20] Granic A, Mendonça N, Sayer AA (2020). Effects of dietary patterns and low protein intake on sarcopenia risk in the very old: the Newcastle 85+ study. Clin Nutr.

[21] Eikelboom JW, Wallentin L, Connolly SJ (2011). Risk of bleeding with 2 doses of dabigatran compared with warfarin in older and younger patients with atrial fibrillation: an analysis of the randomized evaluation of long-term anticoagulant therapy (RE-LY) trial. Circulation.

[22] Halvorsen S, Atar D, Yang H (2014). Efficacy and safety of apixaban compared with warfarin according to age for stroke prevention in atrial fibrillation: observations from the ARISTOTLE trial. Eur Heart J.

[23] Halperin JL, Hankey GJ, Wojdyla DM (2014). Efficacy and safety of rivaroxaban compared with warfarin among elderly patients with nonvalvular atrial fibrillation in the Rivaroxaban Once Daily, Oral, Direct Factor Xa Inhibition Compared With Vitamin K Antagonism for Prevention of Stroke and Embolism Trial in Atrial Fibrillation (ROCKET AF). Circulation.

[24] Graham DJ, Reichman ME, Wernecke M (2015). Cardiovascular, bleeding, and mortality risks in elderly Medicare patients treated with dabigatran or warfarin for nonvalvular atrial fibrillation. Circulation.

[25] Kato ET, Giugliano RP, Ruff CT (2016). Efficacy and safety of edoxaban in elderly patients with atrial fibrillation in the ENGAGE AF-TIMI 48 trial. J Am Heart Assoc.

[26] Steffel J, Giugliano RP, Braunwald E (2016). Edoxaban versus warfarin in atrial fibrillation patients at risk of falling: ENGAGE AF-TIMI 48 analysis. J Am Coll Cardiol.

[27] Amin A, Keshishian A, Dina O (2019). Comparative clinical outcomes between direct oral anticoagulants and warfarin among elderly patients with non-valvular atrial fibrillation in the CMS medicare population. J Thromb Thrombolysis.

[28] Mentias A, Heller E, Vaughan Sarrazin M (2020). Comparative effectiveness of rivaroxaban, apixaban, and warfarin in atrial fibrillation patients with polypharmacy. Stroke.

[29] Lin KJ, Singer DE, Bykov K, Bessette LG, Mastrorilli JM, Cervone A, Kim DH (2023). Comparative effectiveness and safety of oral anticoagulants by dementia status in older patients with atrial fibrillation. JAMA Netw Open.

[30] Yildirim M, Milles BR, Hund H (2025). Outcomes and disease management in patients with atrial fibrillation ≥80 years: data from a consecutive 11-year real-world registry. J Am Heart Assoc.

[31] Nejadghaderi SA, Balibegloo M, Rezaei N (2024). The Cochrane risk of bias assessment tool 2 (RoB 2) versus the original RoB: a perspective on the pros and cons. Health Sci Rep.

[32] Luchini C, Stubbs B, Solmi M, Veronese N (2017). Assessing the quality of studies in meta-analyses: advantages and limitations of the Newcastle Ottawa Scale. World Journal of Meta-Analysis.

[33] Lin L, Chu H, Murad MH, Hong C, Qu Z, Cole SR, Chen Y (2018). Empirical comparison of publication bias tests in meta-analysis. J Gen Intern Med.

[34] Zhang Z, Kossmeier M, Tran US, Voracek M, Zhang H (2017). Rainforest plots for the presentation of patient-subgroup analysis in clinical trials. Ann Transl Med.

[35] Favorito LA (2023). Systematic review and metanalysis in urology: how to interpret the forest plot. Int Braz J Urol.

[36] Feczko E, Fair DA (2020). Methods and challenges for assessing heterogeneity. Biol Psychiatry.

[37] Iwashyna TJ, Burke JF, Sussman JB, Prescott HC, Hayward RA, Angus DC (2015). Implications of heterogeneity of treatment effect for reporting and analysis of randomized trials in critical care. Am J Respir Crit Care Med.

[38] McLaughlin J, Han G, Schalper KA (2016). Quantitative assessment of the heterogeneity of PD-L1 expression in non-small-cell lung cancer. JAMA Oncol.

[39] Richardson M, Garner P, Donegan S (2019). Interpretation of subgroup analyses in systematic reviews: a tutorial. Clin Epidemiol Glob Health.

[40] Wang X, Piantadosi S, Le-Rademacher J, Mandrekar SJ (2021). Statistical considerations for subgroup analyses. J Thorac Oncol.

[41] Chao TF, Chan YH, Chiang CE (2022). Stroke prevention with direct oral anticoagulants in high-risk elderly atrial fibrillation patients at increased bleeding risk. Eur Heart J Qual Care Clin Outcomes.

[42] Deng K, Cheng J, Rao S, Xu H, Li L, Gao Y (2020). Efficacy and safety of direct oral anticoagulants in elderly patients with atrial fibrillation: a network meta-analysis. Front Med (Lausanne).

[43] Yamaji H, Higashiya S, Murakami T (2019). Effects of oral anticoagulants on patients with atrial fibrillation aged 90 years and older: comparison among direct oral anticoagulant, warfarin anticoagulant, and nonanticoagulation. J Cardiovasc Pharmacol.

[44] Mitchell A, Snowball J, Welsh TJ, Watson MC, McGrogan A (2021). Prescribing of direct oral anticoagulants and warfarin to older people with atrial fibrillation in UK general practice: a cohort study. BMC Med.

[45] Lin DS, Lo HY, Huang KC, Lin TT, Lee JK (2023). Efficacy and safety of direct oral anticoagulants for stroke prevention in older patients with atrial fibrillation: a network meta-analysis of randomized controlled trials. J Am Heart Assoc.

